# Eugenol: In Vitro and In Ovo Assessment to Explore Cytotoxic Effects on Osteosarcoma and Oropharyngeal Cancer Cells

**DOI:** 10.3390/plants12203549

**Published:** 2023-10-12

**Authors:** Robert-Cosmin Racea, Ioana-Gabriela Macasoi, Stefania Dinu, Iulia Pinzaru, Iasmina Marcovici, Cristina Dehelean, Laura-Cristina Rusu, Doina Chioran, Mircea Rivis, Roxana Buzatu

**Affiliations:** 1 Faculty of Dental Medicine, “Victor Babeș” University of Medicine and Pharmacy from Timisoara, 9 Revolutiei 1989 Ave., 300070 Timisoara, Romania; robert.racea@gmail.com (R.-C.R.); laura.rusu@umft.ro (L.-C.R.); chioran.doina@umft.ro (D.C.); rivis.mircea@umft.ro (M.R.); roxana.buzatu@umft.ro (R.B.); 2 Multidisciplinary Center for Research, Evaluation, Diagnosis and Therapies in Oral Medicine, “Victor Babeș” University of Medicine and Pharmacy from Timisoara, Eftimie Murgu Square, No. 2, 300041 Timisoara, Romania; 3 Faculty of Pharmacy, “Victor Babes” University of Medicine and Pharmacy from Timisoara, Eftimie Murgu Square No. 2, 300041 Timisoara, Romania; macasoi.ioana@umft.ro (I.-G.M.); iuliapinzaru@umft.ro (I.P.); iasmina.marcovici@umft.ro (I.M.); cadehelean@umft.ro (C.D.); 4 Research Center for Pharmaco-Toxicological Evaluations, Faculty of Pharmacy, “Victor Babeș” University of Medicine and Pharmacy from Timisoara, Eftimie Murgu Square No. 2, 300041 Timisoara, Romania; 5 Pediatric Dentistry Research Center, Faculty of Dental Medicine, “Victor Babeș” University of Medicine and Pharmacy from Timisoara, 9 No., Revolutiei Bv., 300041 Timisoara, Romania

**Keywords:** eugenol, osteosarcoma, oropharyngeal carcinoma, apoptosis, chorioallantoic membrane

## Abstract

Cancer is a significant health problem worldwide; consequently, new therapeutic alternatives are being investigated, including those found in the vegetable kingdom. Eugenol (Eug) has attracted attention for its therapeutic properties, especially in stomatology. The purpose of this study was to investigate the cytotoxicity of Eug, in vitro, on osteosarcoma (SAOS-2) and oropharyngeal squamous cancer (Detroit-562) cells, as well as its potential irritant effect in ovo at the level of the chorioallantoic membrane (CAM). The data obtained following a 72 h Eug treatment highlighted the reduction in cell viability up to 41% in SAOS-2 cells and up to 37% in Detroit-562 cells, respectively. The apoptotic-like effect of Eug was indicated by the changes in cell morphology and nuclear aspect; the increase in caspase-3/7, -8 and -9 activity; the elevated expression of Bax and Bad genes; and the increase in luminescence signal (indicating phosphatidylserine externalization) that preceded the increase in fluorescence signal (indicating the compromise of membrane integrity). Regarding the vascular effects, slight signs of coagulation and vascular lysis were observed, with an irritation score of 1.69 for Eug 1 mM. Based on these results, the efficiency of Eug in cancer treatment is yet to be clarified.

## 1. Introduction

In the history of mankind, medicinal plants have played a significant role in the development of civilizations as well as modern medicine, as evidenced in the traditional remedies that still rely on compounds derived from plants. Furthermore, the discovery of bioactive compounds of natural origin has directly contributed to many of the advances made in the field of cancer [1]. There is ample evidence to support the effectiveness of compounds of natural origin, as many of the chemotherapeutic drugs approved in the past 30 years have their origins in plants [2]. Over the past few years, essential oils derived from plants and aromatic herbs have received considerable attention [3]. Even though research on the antitumor potential of essential oils and their constituents is relatively new, it has been demonstrated that a number of mechanisms are involved, including the direct targeting of tumor cells and interaction with the cellular environment [4]. The monoterpene eugenol (Eug), derived from basil, cloves, and cinnamon, is an essential component of aromatic oils. In studies of Eug’s biological effects, it was found to be effective as an anti-inflammatory, analgesic, antiviral and antioxidant [5,6,7,8]. These properties have primarily led to Eug’s use in stomatology [9]. Furthermore, Eug has demonstrated versatility as a therapeutic molecule, and is currently being investigated for its antitumor properties, both as a molecule and as a derivative [10]. In vitro and in vivo studies conducted on Eug have demonstrated that it is capable of interfering with a variety of essential cellular functions, including triggering apoptosis and cell-cycle arrest [11]. Nevertheless, the mechanism of action of Eug may vary depending on the type of cancer being treated. Eug has proven useful in treating cancers such as melanoma, leukemia, or gastric cancer [12]. A number of mechanisms contribute to the antitumor activity of this compound, such as inhibiting cell proliferation, migrating, and interfering with angiogenesis; however, many mechanisms remain unclear [9].

Osteosarcoma (OS) is a primary malignant tumor of the bones that occurs more frequently in adolescents and adults over 60 years of age [13]. While osteosarcoma is considered a rare malignancy, it is the most common primary bone malignancy among children and adolescents, with a frequency of approximately 3% in European countries [14]. OS is typically sporadic, but several factors can contribute to its initiation and progression, including genetic predisposition, Paget disease, or radiotherapy and chemotherapy [15,16,17]. From a histopathological standpoint, the neoplasm is characterized by aggressive, osteoid-producing or immature malignant mesenchymal cells [18]. Generally, OS occurs at the metaphyses of long tubular bones, in particular the proximal humerus or femur, but it may also occur in other areas such as the spine or skull [13]. As a rare form of OS, osteosarcoma of the jaw accounts for 7% of all osteosarcomas and 1% of all malignant diseases of the head and neck. Without the appropriate treatment, this malignancy has an unfavorable prognosis [19]. Surgical treatment is one of the main therapeutic options, but it can be a challenging process due to the complex anatomy of the maxillo-facial region. To prevent amputation and increase survival rates by about five years, surgery is usually combined with other types of treatment. There are a variety of treatment methods available to patients, including chemotherapy, radiation therapy, immunotherapy, and more recently, gene therapy [20]. OS treatment has improved patient outcomes; however, it is still limited, particularly in patients with metastases or patients who have developed resistance to chemotherapy [21]. Natural compounds have shown promising therapeutic effects in the treatment of osteosarcoma, accompanied by few side effects and low cost. Phytochemicals such as curcumin, genistein, resveratrol, etc., are being studied for their potential therapeutic effects on OS [2]. In addition to these compounds, Eug was shown to have a potent effect on inhibiting the proliferation of osteosarcoma cells by inducing apoptosis [12].

Oropharyngeal squamous cell carcinoma (OPSCC), also referred to as throat or tonsil cancer, represents a continuous public health problem worldwide due to its alarming increase in incidence in both young and old populations [22,23]. OPSCC develops from the non-keratinizing stratified mucosal epithelium that is present in the upper aerodigestive tract [12], being localized in the middle part of the pharynx, called the oropharynx, which includes several regions, such as the tonsils, soft palate, and tongue. OPSCC represents the main type of malignancy originating in this region, the majority of oropharyngeal cancers (over 90%) being squamous cell neoplasms [22]. From an etiological perspective, OPSCC development is usually related to tobacco and alcohol consumption; however, another factor that was widely associated with OPSCC is the infection with the human papillomavirus (HPV) virus [24]. The current treatment options for OPSCC resort to surgical intervention and radio- or chemoradiotherapy, which were associated with several risks and complications, including tissue damage and toxicity occurring especially in the head and neck areas, altered speech or swallowing, and a high risk for the development of secondary cancers. In addition, the 5-year survival rate is approximately 50%. Considering the poor prognosis and the need to minimize adverse effects, especially among young patients, it is essential to find new therapeutic alternatives [25]. A variety of phytochemicals are effective in the treatment of oropharyngeal cancer through multiple mechanisms, including inhibition of metastasis, angiogenesis, and cell-cycle arrest. Studies have explored the potential role that several natural compounds may play in treating oral cancer, including curcumin, quercetin, and genistein [26]. In addition, Eug was shown to be effective in reducing the proliferation and invasion of oral cancer cells through the induction of apoptosis [27].

In light of these scientific data, the current study is aimed at a comparative in vitro assessment of the antitumor potential of Eug, a natural compound commonly used in dental practice, against two particular cancer types developing in the head and neck area—osteosarcoma, which can affect the oral bones, and oropharynx squamous carcinoma. The present research emphasizes the cytotoxic effect of Eug on SAOS-2 and Detroit-562 cell lines, describing the cell-death mechanism underlying its antitumor effect in vitro. The investigation of the potential toxicity of Eug at the vascular level was also of interest for this study.

## 2. Results

### 2.1. Evaluation of the Cytotoxic Profile

**Figure 1:**
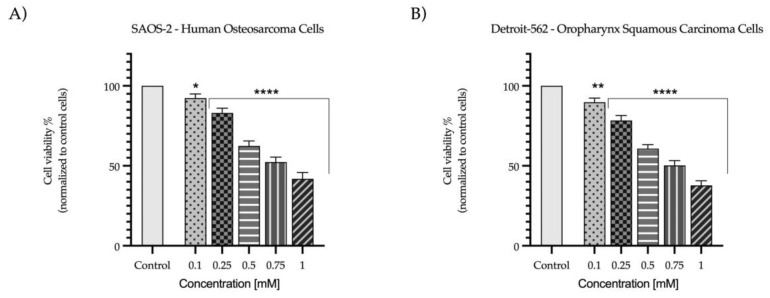
In vitro cell viability evaluation of Eug (0.1, 0.25, 0.5, 0.75, and 1 mM) in: (**A**) SAOS-2 (human osteosarcoma cells) and (**B**) Detroit-562 (oropharynx squamous carcinoma cells). An MTT colorimetric assay was performed after 72 h treatment. The results are presented as viability percentages (%) normalized to control (unstimulated cells). The presented data are expressed as mean values ± SD of three independent experiments performed in triplicate. For analyzing the statistical differences between the control and treated groups, one-way ANOVA test was used, followed by a Dunnett’s multiple comparison post hoc test. “*” marks the statistical differences between data (* *p* < 0.05; ** *p* < 0.01; and **** *p* < 0.0001).

To investigate the potential in vitro antitumor activity of Eug on SAOS-2 and Detroit-562 cell lines, an MTT assay was performed at the end of the 72 h treatment. According to the results (Figure 1), Eug exerted a concentration-dependent cytotoxicity against both types of cancer cells. Compared to the control (representing untreated cells), a significant reduction in the viability of SAOS-2 (92%) and Detroit-562 (89%) cells was obtained at the lowest tested concentration (0.1 mM); however, the highest cytotoxic effect was recorded at 1 mM, when the viability percentages were lowered to 41% (SAOS-2), and 37% (Detroit-562), respectively.

**Figure 2:**
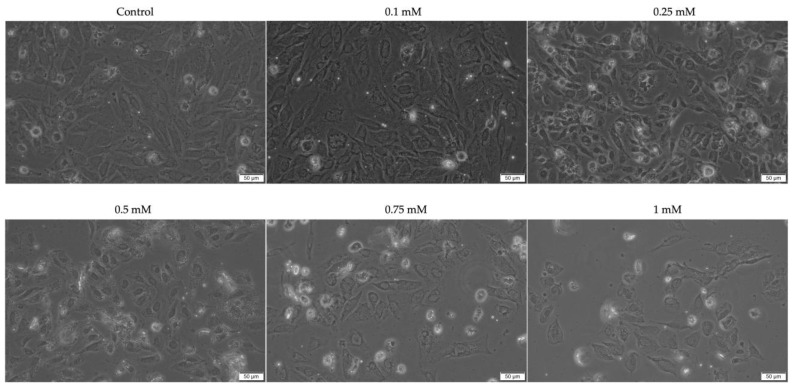
In vitro morphological changes of SAOS-2 cells after 72 h of stimulation with Eug (0.1, 0.25, 0.5, 0.75, and 1 mM). The pictures were taken at a magnification of 20×, and the scale bar indicates 50 μm.

**Figure 3:**
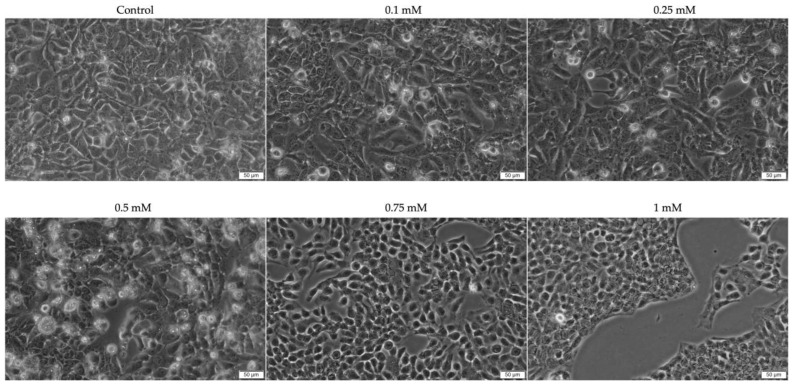
In vitro morphological changes of Detroit-562 cells after 72 h of stimulation with Eug (0.1, 0.25, 0.5, 0.75, and 1 mM). The pictures were taken at a magnification of 20×, and the scale bar indicates 50 μm.

To further explore the cytotoxicity of Eug, the morphology and confluence of SAOS-2 and Detroit-562 cells were evaluated at 72 h post-stimulation. In the case of SAOS-2 cells (Figure 2), a significant impact of Eug on the cellular shape (i.e., shrinkage and rounding leading to detachment from the plate and floating) and confluence was observed at concentrations higher than 0.1 mM, the most noticeable morphological changes being induced at the concentration of 1 mM. Similarly, Eug 0.1 mM generated no significant changes in the aspect or confluence of Detroit-562 cells compared to the control; however, at higher concentrations, a dose-dependent decrease in the cells’ confluence was noticed (Figure 3). At 0.5 mM, Eug caused a visible increase in the number of round, floating cells compared to the control or lower concentrations. Moreover, the Detroit-562 cells treated with Eug 0.75 and 1 mM appeared stressed, shrunk, and round but were not detached or floating.

### 2.2. Detection and Quantification of Nuclear Morphology

**Figure 4:**
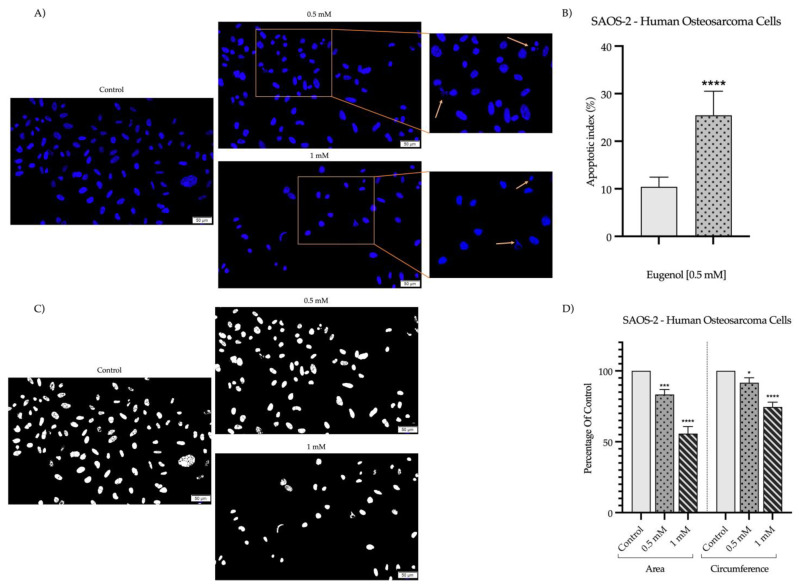
(**A**) SAOS-2 nuclei stained with DAPI after 72 h treatment with Eug (0.5 and 1 mM). The yellow arrows indicate signs of apoptosis. The scale bars represent 100 µm. (**B**) Apoptotic index (AI) determination in DAPI-stained SAOS-2 cells following 72 h treatment with 0.5 mM Eug. (**C**) ImageJ analyses of nuclear morphology. (**D**) As compared to control cells, Eug stimulation (0.5 and 1 mM) led to a decrease in the area and circumference of the nuclei. The data are expressed as mean values ± SD of three independent experiments performed in triplicate For analyzing the statistical differences between the control and treated groups, one-way ANOVA test was used, followed by a Dunnett’s multiple comparison post hoc test. “*” marks the statistical differences between data (* *p* < 0.05; *** *p* < 0.001; **** *p* < 0.0001).

**Figure 5:**
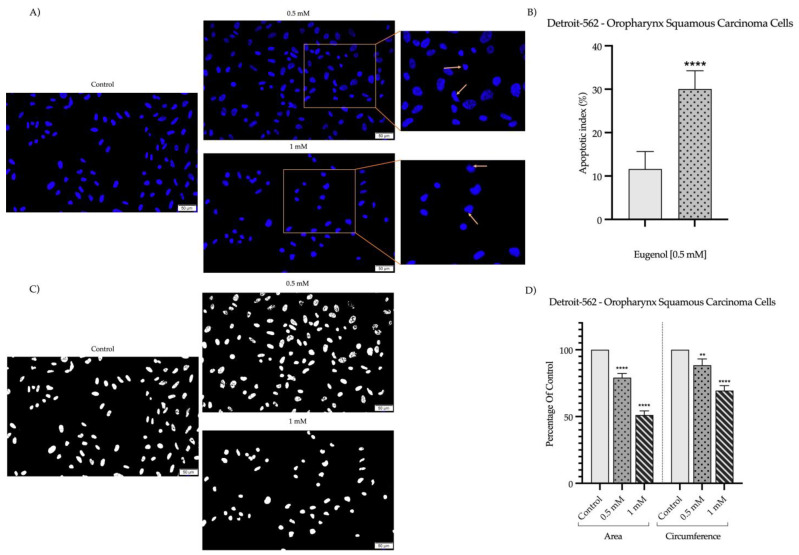
(**A**) Detroit-562 nuclei stained with DAPI after 72 h treatment with Eug (0.5 and 1 mM). The yellow arrows indicate signs of apoptosis. The scale bars represent 100 µm. (**B**) Apoptotic index (AI) determination in DAPI-stained Detroit-562 cells following 72 h treatment with 0.5 mM Eug. (**C**) ImageJ analyses of nuclear morphology. (**D**) As compared to control cells, Eug stimulation (0.5 and 1 mM) led to a decrease in the area and circumference of the nuclei. The data are expressed as mean values ± SD of three independent experiments performed in triplicate For analyzing the statistical differences between the control and treated groups, one-way ANOVA test was used, followed by a Dunnett’s multiple comparison post hoc test. “*” marks the statistical differences between data (** *p* < 0.01; **** *p* < 0.0001).

To evaluate the potential cell-death mechanism related to the cytotoxicity of Eug on SAOS-2 and Detroit-562 cancer cells, DAPI staining was performed to highlight the morphological changes occurring at the nuclear level at 72 h post-treatment. As shown in Figure 4A and Figure 5A, respectively, the nuclei of the control cells are uniformly stained in blue, presenting a specific oval or round shape. Comparatively, the nuclei of the SAOS-2 and Detroit-562 cells exposed to Eug (0.5 and 1 mM) presented an apoptotic-like appearance, being fragmented and massively condensed. Consequently, a significant elevation in the apoptotic index (to 25% for SAOS-2 cells and 30% for Detroit-562 cells) was determined compared to the control (Figure 4B and Figure 5B), indicating an increase in the percentage of apoptotic nuclei following the 72 h exposure to Eug 0.5 mM. Additionally, both the nucleus’ circumference and area decreased significantly following treatment with Eug (Figure 4C,D and Figure 5C,D).

### 2.3. Real Time PCR Study and Caspases Activity

**Figure 6:**
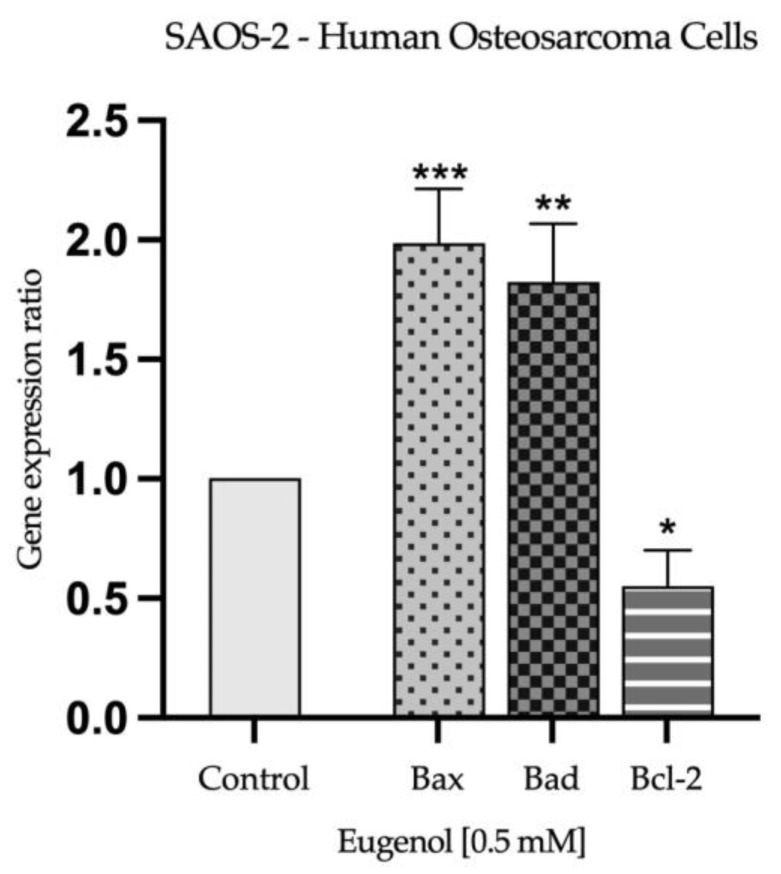
Relative fold change in expression of mRNA of pro-apoptotic (Bax and Bad) and anti-apoptotic (Bcl-2) markers in osteosarcoma cells (SAOS-2), 72 h after exposure to Eug 0.5 mM. mRNA expression levels normalized to 18S expression. Mean values ± SD of three independent experiments are presented. One-way ANOVA with Dunnett’s multiple comparisons post hoc test were used to identify the statistical differences between data (* *p* < 0.05; ** *p* < 0.01 and *** *p* < 0.001).

**Figure 7:**
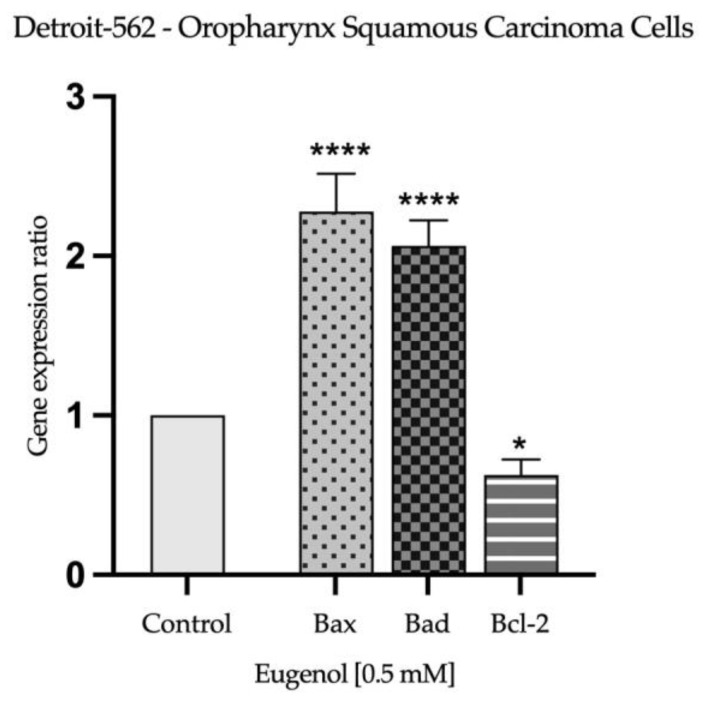
Relative fold change in expression of mRNA of pro-apoptotic (Bax and Bad) and anti-apoptotic (Bcl-2) markers in oropharynx squamous carcinoma cells (Detroit-562), 72 h after exposure to Eug 0.5 mM. mRNA expression levels normalized to 18S expression. Mean values ± SD of three independent experiments are presented. One-way ANOVA with Dunnett’s multiple comparisons post hoc test were used to identify the statistical differences between data (* *p* < 0.05; and **** *p* < 0.0001).

Based on the observations made during the evaluation of nuclear morphology, to further explore the potential apoptotic effect induced by Eug 0.5 mM on SAOS-2 and Detroit-562 cells after 72 h of stimulation, its impact on the expression of apoptosis-related genes was determined. As presented in Figure 6 and Figure 7, Eug exerted a similar effect in both tested cells lines by significantly increasing the expression of pro-apoptotic markers Bax and Bad, while decreasing the expression of the anti-apoptotic marker Bcl-2. 

**Figure 8:**
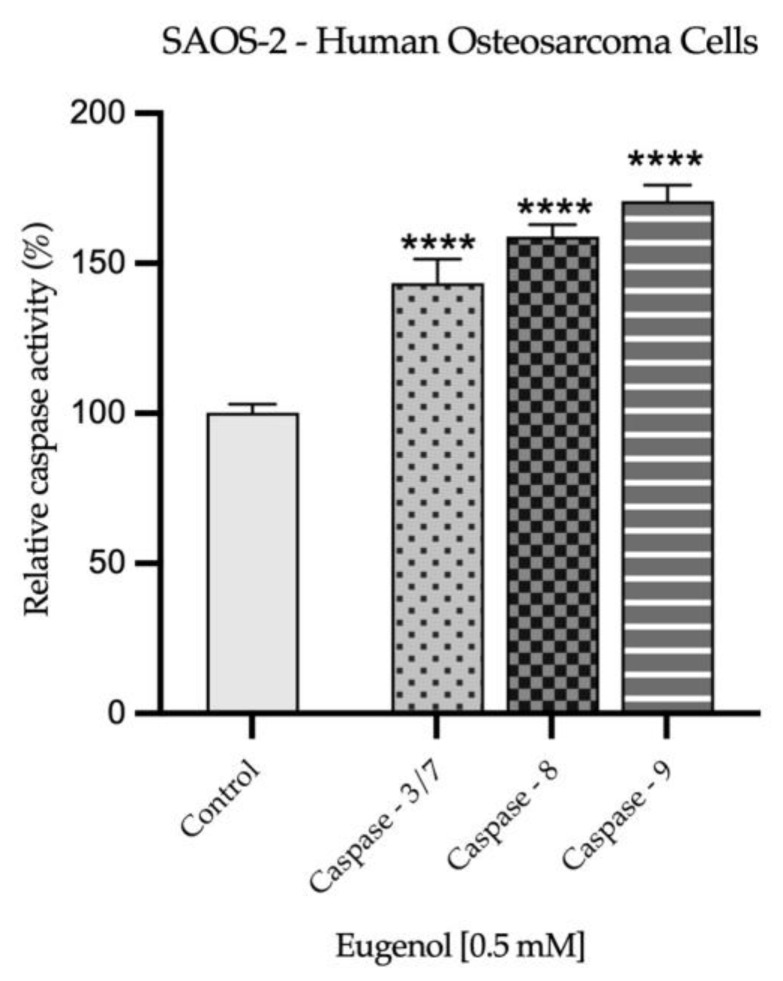
Activity of caspases 3/7, 8, and 9 in SAOS-2 cells, 72 h after exposure to Eug 0.5 mM. The data are presented as mean ± SD of the three independent experiments, in reference to the non-exposure group. One-way ANOVA and Dunnett’s post hoc test were used to identify the statistical differences (**** *p* < 0.0001).

**Figure 9:**
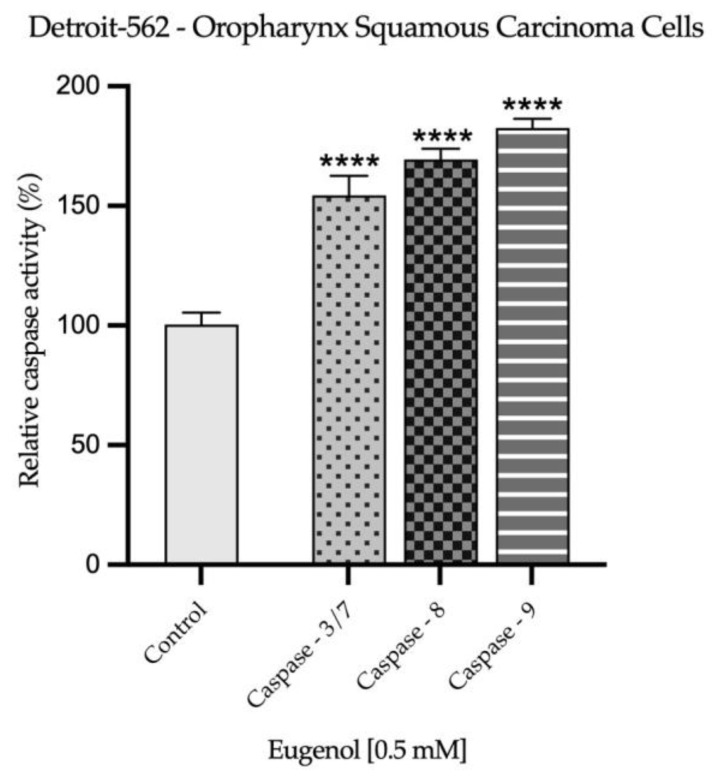
Activity of caspases 3/7, 8, and 9 in Detroit-562 cells, 72 h after exposure to Eug 0.5 mM. The data are presented as mean ± SD of the three independent experiments, in reference to the non-exposure group. One-way ANOVA and Dunnett’s post hoc test were used to identify the statistical differences (**** *p* < 0.0001).

The influence of Eug 0.5 mM on the activity of three caspases (3/7, 8, 9) was assessed after the 72 h treatment of SAOS-2 and Detroit-562 cells. The results, presented in Figure 8 and Figure 9, describe a significant increase in the activation of all investigated caspases upon the cells’ exposure to Eug 0.5 mM and are in accordance with the previous results, suggesting the pro-apoptotic effect of Eug at this concentration in the tested cancer cell lines. 

### 2.4. The RealTime-Glo™ Annexin V Apoptosis and Necrosis Assay 

**Figure 10:**
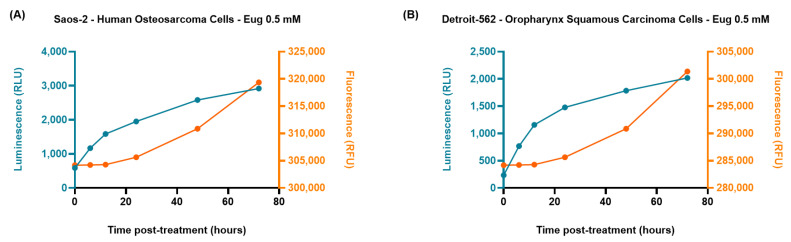
Saos-2 (**A**) and Detroit-562 (**B**) cells were treated with Eug 0.5 mM and the RealTime-Glo™ Annexin V Apoptosis/Necrosis Assay reagent. Luminescence (corresponding to phosphatidylserine externalization) and fluorescence (corresponding to loss of cell membrane integrity) signals were measured at different time points (0, 6, 12, 24, 48, and 72 h). RLU = relative light units; RFU = relative fluorescence units.

The ability of Eug 0.5 mM to induce apoptosis in SAOS-2 and Detroit-562 cancer cells was also evaluated by applying a kinetic annexin-based method. As presented in Figure 10, the treatment with Eug 0.5 mM induced a time-dependent increase in the luminescence signal (indicating phosphatidylserine externalization), which preceded the increase in fluorescence signal (indicating membrane integrity loss due to secondary necrosis), thus, showing an apoptotic response in both treated cell lines following treatment.

### 2.5. Hen’s Egg Test—Chorioallantoic Membrane (HET-CAM)

**Figure 11:**
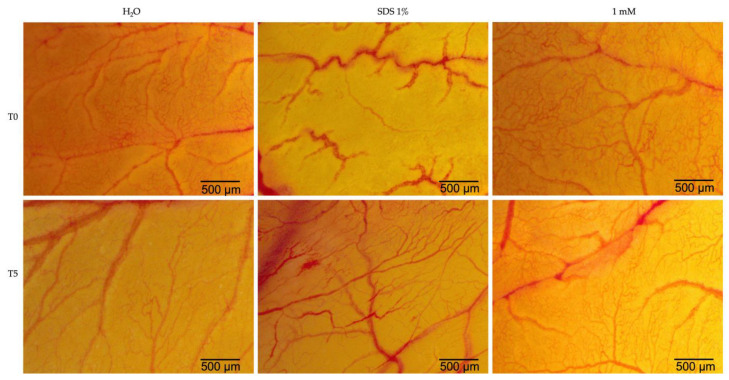
Analysis of the irritant potential of eugenol at a concentration of 1 mM by the HET-CAM method. Stereomicroscopic images of CAMs inoculated with negative control—H_2_O, positive control—SDS 1%, and test compound—Eug 1 mM achieved at time 0 (T0) and 5 min after application (T5).

Eug was evaluated for its irritant potential using the chorioallantoic membrane of hen’s eggs at a concentration of 1 mM, the highest concentration tested in this study. In order to obtain a more precise evaluation, water was used as a negative control, and sodium dodecyl sulfate (SDS) 1% solution was used as a positive control. Eug caused vascular lysis and coagulation at the vascular level but only to a limited extent (Figure 11). In Table 1, the irritation scores obtained for the two types of controls and eugenol are shown. Water was recorded as having the lowest irritation score, whereas SDS 1% showed the highest irritation score. Eug, on the other hand, presented an irritation score of 1.69, indicating a low potential for irritation.

## 3. Discussion

In spite of the immense efforts that have been made in this field, cancer continues to pose a global health burden, with both the number of newly diagnosed cases and the number of deaths increasing over time. Researchers are currently investigating therapeutic approaches to cancer, which is a relatively new area of research [28]. Osteosarcoma is one of the most common types of bone cancer, affecting primarily the long tubular layers of the bone [13]. Even though it is not common, there have also been reports of facial bones being damaged, particularly in the jaw. It is important to note that this type of neoplasm presents two major difficulties. First, there is the increased incidence among adolescents, and subsequently, there is the high degree of metastasis, especially at the lung level, and the reduction in the survival rate [29]. Oropharyngeal squamous cell carcinoma is commonly referred to as throat cancer. The threat posed by this type of cancer is due to the fact that it can spread through the blood and lymphatic system, resulting in metastases. Human papillomavirus (HPV)-positive oropharyngeal carcinomas have been on the rise in recent years. Although this type of cancer responds well to current therapies, efforts have been made to find new therapies that are less toxic [30]. Recent botanical and pharmaceutical studies have suggested that medicinal plants may be effective as antitumor therapeutic agents. A significant challenge in the research of natural compounds in cancer treatment is the assessment of their toxicological profile as well, as the clarification of their biological mechanism of action [31]. Eug (4-alil-2-metoxifenol) is a phenylpropanoid compound that can be found in a range of plants, including holy basil, cloves, turmeric, and ginger. *Eugenia caryophyllata* contains the highest proportion of Eug; however, its extraction is costly. In order to overcome this limitation, other plants that are more cost-effective are used, such as ginger or laurel [32]. There is a wide variety of biological actions associated with Eug, which justify its use in industry and medicine at present. Aside from its antioxidant properties, Eug also has anti-inflammatory and local anesthetic properties, which is why it is utilized in dental practice [33]. In view of Eug’s wide spectrum of applications, the present study evaluated its antitumor properties at the level of osteosarcoma and oropharyngeal squamous cell carcinoma cells in vitro. Additionally, Eug’s irritant potential for the vascular plexus was evaluated by using a hen’s egg chorioallantoic membrane as a biological model.

Thus, the first step of the study consisted of evaluating the cytotoxic effects of five Eug concentrations ranging between 0.1 and 1 mM on osteosarcoma cells—SAOS-2 and oropharyngeal carcinoma cells—Detroit-562 after 72 h of stimulation. Based on the results, both types of cells were affected in a dose-dependent manner by Eug treatment, with cell viability decreasing up to 41% in the case of SAOS-2 cells and up to 37% in the case of Detroit-562 cells, at the concentration of 1 mM. The concentrations of Eug used in this study were selected based on a thorough review of the literature [34,35,36,37]. In addition, the present research represents a continuation of two previous studies, in which the cytotoxic potential of Eug was evaluated using healthy human cells—gingival fibroblasts (HGF) and keratinocytes (HaCaT) and tumor cells—tongue squamous carcinoma cells (SCC4), and colorectal carcinoma cells (HCT-116) [38,39]. A dose-dependent decrease in cell viability was observed in tongue squamous carcinoma cells when Eug was administered at concentrations between 0.1 and 1 mM [38]. A similar trend was also observed in the case of colorectal carcinoma cells—HCT-116, which, when exposed to concentrations between 0.1 and 1 mM of Eug, showed a decrease of up to 34% in cell viability [39]. In terms of Eug’s potential cytotoxicity on healthy cells, it was previously tested on gingival fibroblasts and keratinocytes at the same concentration and time interval as was used in the current study. Cell viability did not decrease more than 70% in either of the two cell lines studied [38,39]. The ISO Standard 10993-5:2009 defines a substance as potentially toxic if it reduces cell viability by at least 30% [40]. In this regard, Eug at concentrations up to 1 mM is not considered cytotoxic on healthy human cells. Shin et al. investigated the cytotoxicity of Eug on an osteosarcoma cell line—HOS, finding that Eug treatment causes a concentration- and time-dependent decrease in cell viability, comparable to the results of the present study [37]. Additionally, Razak et al. illustrated that the conjugation of Eug with D-glucose potentiates the cytotoxic effect in osteosarcoma cells—K7M2 [41]. Among the conventional antitumor therapies approved by the FDA for the treatment of osteosarcoma and pharyngeal cancer are methotrexate and cetuximab [42,43]. According to our previous studies, the effects of these two antitumor substances were evaluated at the level of non-tumor cells—HaCaT. Based on the results of the study, both compounds reduced cell viability in a dose-dependent manner, although methotrexate showed a more pronounced cytotoxic response [44]. In contrast to our previous studies on the cytotoxic effects of Eug on healthy cells, HaCaT and HGF, methotrexate and cetuximab exhibit an even more dramatic reduction in cell viability [38,39]. Sramek and colleagues evaluated the effects of methotrexate on SAOS-2 cells, and the results indicated that high concentrations of 100 μM significantly reduced cell viability to below 20% [45]. A further study conducted by Ali Khyavi et al. investigated the effects of methotrexate on SAOS-2 cells, and the results suggested that the drug possesses a pronounced cytotoxic effect [46]. The team led by Young evaluated the potency of cetuximab as a cytotoxic agent in Detroit-562 cells. According to their findings, cetuximab decreased cell viability, but its effectiveness was not as great as that of afatinib [47]. By contrast, Eug tested in the present study exerted a considerable cytotoxic effect, but it was not as intense as the two antitumor substances. Eug, however, caused a weak reduction in cell viability at the level of healthy cells, indicating a lower cytotoxic potential than conventional antitumor agents. In the light of these findings, natural compounds have become a hot topic in antitumor research, with studies demonstrating that they can reduce chemotherapy drug toxicity while simultaneously enhancing therapeutic efficacy [48].

In a similar study, Duan and colleagues examined the cytotoxic potential of Eug in oral squamous cell carcinoma (SCC9). The results indicated that concentrations similar to those used in the present study reduced the viability and proliferation of tumor cells [49]. Eug was also found to reduce cell viability in oropharyngeal carcinoma cells by causing cell apoptosis when tested at concentrations up to 2 mM [50]. In the literature, Eug is described as possessing antitumor properties at the level of various types of cell lines, as well as suggesting possible mechanisms of action associated with these properties. Therefore, Eug induces apoptosis in cells via a mechanism that is dependent upon mitochondrial function and the production of reactive oxygen species [51]. It was also observed that the antioxidant effect was associated with the decrease in inflammatory cytokines, as well as the induction of cell-cycle arrest [52,53]. In addition, the research suggests that the combination of Eug with conventional chemotherapeutics, such as cisplatin, may increase the sensitivity of tumor cells to the treatment [54].

It is well documented that alterations in cell morphology have played a significant role in characterizing the type of cell death and assessing the cytotoxicity of various compounds over the past century [55]. In the development of new therapeutic agents, the term “cytotoxicity” has a complex meaning. A compound is considered cytotoxic in monolayer cell cultures if it interferes with different processes such as cell attachment or growth, as well as cell morphology [56]. Therefore, the next step of the study involved evaluating Eug’s effect on cellular morphology. Based on the results, both types of cell lines studied showed a dose-dependent response. As a result of the 1 mM concentration, the most intense morphological changes were noticed, including rounding of cells, their detachment from the plate, cell shrinkage, the loss of connections with neighboring cells, and a decrease in the confluency of the cells. The first step in distinguishing healthy cells from necrotic or apoptotic cells is to examine the morphology of the cells. Cellular rounding and contraction, as well as membrane blebbing, are major characteristics of apoptotic cells [57]. Based on the results of the present study, these modifications were observed and described. A variety of these effects were previously reported in relation to colorectal cancer and squamous cell carcinoma of the tongue [38,39] The studies conducted on other types of tumor cells treated with Eug showed similar morphological alterations. The results of Vidhya’s study demonstrated that the treatment of MCF-7 breast cancer cells with Eug results in morphological changes characteristic of cell apoptosis [58]. Furthermore, cervical cancer cells—HeLa, breast cancer cells—MCF-7 and MDA-MB-231 or melanoma cells—SK-Mel-31 showed apoptotic characteristic after treatment with Eug [34,59].

In addition to damage in cell morphology, apoptosis is characterized by alterations to nuclear structures. A powerful technique for identifying apoptotic changes at the nuclear level is fluorescence microscopy [60]. During apoptosis, nuclear fragmentation and chromatin condensation are among the most significant transformation at the level of cell nuclei. The first phase of condensation occurs at the periphery of the nuclei, forming a crescent-shaped structure. Chromatin condensation can be visualized using different techniques involving dyes that bind to DNA (e.g., DAPI) [55]. A DAPI staining was performed in the present study in order to visualize the nuclei of SAOS-2 and Detroit-562 cells. At a concentration of 0.5 mM, Eug induces the first signs of nuclear condensation, whereas at a concentration of 1 mM, chromatin condensation, and nuclear fragmentation are observed. Furthermore, Eug was found to induce similar impair in the nuclei of tumor cells in both squamous cancers of the tongue and colorectal carcinomas, whereas healthy cells showed no significant changes in the nuclear aspect [38,39]. Previous studies have also indicated that Eug has similar effects on the nuclei. Consequently, Eug induced chromatin condensation at concentrations up to 200 µM in cervical cancer cells—HeLa [61]. Also, at the level of human melanoma cells—G361, Eug at a concentration of 1 mM induced the inhibition of cell proliferation and apoptosis, which was confirmed by utilizing Hoechst staining to visualize the nuclei, which exhibited similar changes to those observed in the present study [62]. A report by Shin and colleagues indicates that Eug induces apoptosis in osteosarcoma cells, altering their nuclei’s morphology and causing chromatin condensation [37].

Several other damages are discovered during cellular apoptosis in addition to alterations in the nuclei of the cells. Hence, apoptosis is initiated through the inhibition of anti-apoptotic proteins (Bcl-2) by pro-apoptotic proteins, leading to the release of pro-apoptotic effector proteins Bax and Bak. As a result of the release of apoptogenic factors, such as cytochrome c, caspase-9 is activated, followed by caspase-3/7, the executor caspase. As well, the activated form of caspase-8 can induce cell apoptosis by directly activating caspase-3/7 and cleaving the pro-apoptotic protein BH3 Bid [63]. The present study evaluated the effects of Eug on the expression of the anti-apoptotic protein Bcl-2, the pro-apoptotic proteins Bax and Bad, and the activity of caspases 3/7, 8 and 9. At the concentration of 0.5 mM, Eug decreased the expression of the anti-apoptotic protein Bcl-2 and increased the expression of the pro-apoptotic proteins Bax and Bad in both tumor cell lines. The activity of caspases, which play an important role in the initiation and execution of apoptosis, was also significantly increased. The in vitro results obtained with MDA-MB-231, MDA-MB-468, and BT-20 cells demonstrated that Eug induces apoptosis by increasing the Bcl-2/Bax ratio, as well as by increasing the expression of Bax protein and caspases 3 and 9 [64]. Other studies have found that Eug has a pro-apoptotic effect on cervical carcinoma cells by downregulating the expression of the Bcl-2 protein, increasing the expression of Bax, and activating caspase-3 and -9 [54,62,65]. In addition, Eug inhibited the expression of Bcl-2 protein in lung cancer cells. Aside from this, it upregulated the expression of the Bax and p-53 proteins and activated caspase-3 as well [66]. An in vitro study performed on gastric carcinoma cells—AGS demonstrated that Eug induces apoptosis by increasing the expression of caspases 3 and 8 in a p53-independent manner [67]. Additionally, these findings are consistent with our prior studies investigating Eug’s antitumor potential against squamous cell carcinoma of the tongue and colorectal carcinoma [38,39]. Using caspase inhibitors to elucidate apoptotic pathways is an important and valuable approach in the field of cell biology and molecular biology. Caspases are a family of proteases that play a central role in apoptosis, the programmed cell-death process. By inhibiting caspases, researchers can manipulate and study the apoptotic pathways in greater detail [68]. The present study has made significant progress toward unraveling the complexities of apoptotic pathways involved in the antitumor potential of Eug. As a result of the findings, key molecular events and regulatory factors that drive apoptosis have been identified. It is important to acknowledge, however, that the role of caspases, the central executioners of apoptosis, is still an area ripe for further investigation. Caspase inhibitors, which are not explored in this study, offer an exciting opportunity to gain deeper mechanistic insights into cellular processes. The use of caspase inhibitors emerges as a promising direction for future investigations, offering the potential to unravel further intricacies in apoptotic regulation and pave the way for novel therapeutic strategies.

Although there are a variety of methods available for evaluating the cell-death process in vitro, most of these techniques are resource-intensive and destructive [69]. The current study also employed the RealTime-Glo^TM^ Annexin V Apoptosis and Necrosis assay to explore the cell-death mechanism induced by Eug 0.5 mM in these cell lines following treatment. The choice of this method was based on its advantages, such as (i) real-time monitoring of apoptosis and necrosis in living cells; (ii) double detection (at the same time point) of both cell apoptosis and necrosis, being useful in experiments that study the complex processes of cell death; (iii) high sensitivity, precision and versatility; and (iv) obtainment of kinetic information that allows the temporal characterization of the cell-death processes [70]. The results (Figure 10) indicated that the exposure of SAOS-2 and Detroit-562 cells to Eug led to a delay between the onset of the increasing luminescence signal (representing the externalization of phosphatidylserine) and the onset of the increasing fluorescence signal (representing loss of membrane integrity) which suggests an apoptotic-specific phenotype and confirms the results obtained previously (Figure 4, Figure 5, Figure 6, Figure 7, Figure 8 and Figure 9).

Finally, the potential toxicity of Eug was investigated in ovo. A unique and extremely useful biological model in medical research is the chorioallantoic membrane of the hen’s egg. As a result of the rich vascular network associated with CAM, it is possible to study different substances from a pharmaco-toxicological perspective [71]. According to the calculation of the irritation score, substances can be categorized as follows: (i) nonirritating substances—IS = 0–0.9; (ii) irritating substances—IS = 1–8.9; and (iii) strongly irritating substances—IS = 9–21 [72]. The present study assessed Eug’s irritant potential at the level of the chorioallantoic membrane, observing reactions such as hemorrhage, vascular lysis, and intravascular coagulation. Based on the results obtained in this case, Eug at a concentration of 1 mM exerts mild irritating effects at the level of the vascular plexus, with an irritation score of 1.69. So far as we are aware, Eug has not been tested alone at the level of the chorioallantoic membrane for evaluating the irritant potential on the blood vessels. The findings of this study support previous studies that investigated the antitumor potential of eugenol [38,39]. An earlier study carried out by Ahmad and colleagues evaluated the irritation potential of a nanoformulation containing Eug at the level of the chorioallantoic membrane, achieving a score comparable to the current study [73]. The previous studies have used chorioallantoic membranes as a means of determining the antiangiogenic potential of Eug found in *Cinnamomum tamala* methanolic extract, with encouraging results [74]. Furthermore, the CAM model was used to evaluate the teratogenic potential of Eug, indicating that Eug possessed a clear teratogenic risk for chick embryos [75].

## 4. Materials and Methods

### 4.1. Reagents

The reagents used in the present study: Eugenol, phosphate saline buffer (PBS), fetal calf serum (FCS), penicillin–streptomycin, and MTT [3-(4,5-dimethylthiazol2-yl)-2,5 -diphenyltetrazolium bromide], were purchased from Sigma Aldrich Merck KgaA (Darmstadt, Germany). DAPI (4′,6-diamidino-2-phenylindole) was obtained from Thermo Fisher Scientific (Waltham, MA, USA). The media used for cell culture, Dulbecco’s Modified Eagle’s Medium (DMEM-ATCC^®^ 30–2002™) and Eagle’s Minimum Essential Medium, (EMEM-ATCC^®^ 30-2003™), were provided by ATCC (American Type Culture Collection, Lomianki, Poland). For RT-PCR, the following primers were used: 18S, Bax, Bcl-2 purchased from Thermo Fisher Scientific, Inc. (Waltham, MA, USA), and Bad purchased from Eurogentec (Seraing, Belgium). Caspase-Glo(R) 3/7 Assay (G8090), Caspase-Glo(R) 8 Assay (G8200), Caspase-Glo(R) 9 Assay (G8210), and the RealTime-Glo™ Annexin V Apoptosis and Necrosis Assay (JA1011) were purchased from Promega Corporation (Madison, WI, USA). Cell-culture-appropriate characteristics were present in all reagents.

### 4.2. Cell Culture

The present study was performed on two human cancer cell lines—SAOS-2 (osteosarcoma cells) and Detroit-562 (oropharynx squamous carcinoma cells), respectively, which were received from ATCC in the form of frozen vials. SAOS-2 cells were cultured in DMEM, while Detroit-562 cells were cultured using EMEM. Both media contained 10% FCS and 1% penicillin (100 U/mL)–streptomycin (100 μg/mL) mixture. Over the course of the experiments, the cells were incubated at 37 °C and 5% CO_2_.

### 4.3. Cellular Viability Evaluation

A colorimetric approach—the MTT [3,4,5-dimethylthiazol-2-yl)-2,5-diphenyltetrazolium bromide] assay, was used to determine cell viability. This was accomplished by cultivating the cells in 96-well plates at a density of 1x10^4^ cells per well. Upon achieving a confluence of approximately 90%, the cells were stimulated for 72 h with five concentrations of Eug (0.1; 0.25; 0.5; 0.75 and 1 mM). The Eug solutions were prepared by diluting stock solution in culture medium. After this time interval, 10 μL of MTT reagent was added to each well, and the cells were incubated for three hours at 37 °C. To solubilize the MTT formazan crystals, 100 μL of solubilizer were added to each well, and after 30 min, the absorbance was measured at 570 nm using Cytation 5 (BioTek Instruments Inc., Winooski, VT, USA).

### 4.4. Cellular Morphology Evaluation

As a means of determining the impact of Eug on the morphology of cells, the microscopic evaluation of SAOS-2 and Detroit-562 cells was carried out after exposure for 72 h. The changes were observed under bright field illumination with the help of the Olympus IX73 inverted microscope (Olympus, Tokyo, Japan), and the processing of the images for the purpose of analysis was carried out by means of CellSens Dimensions v.17 Software (Olympus, Tokyo, Japan).

### 4.5. Nuclear Morphology Evaluation

Using immunofluorescence, it was possible to provide further details regarding Eug’s effects on osteosarcoma and oropharynx squamous carcinoma nuclei. The technique was performed for cells stimulated with Eug 0.5 and 1 mM for 72 h. The applied protocol assumed the following stages: (i) culturing the cells in 12-well plates and stimulating them after reaching the desired confluence with Eug 0.5 and 1 mM for 72 h; (ii) washing with PBS and fixing the cells with 4% paraformaldehyde; (iii) permeabilization with Triton X 0.2%; (iv) blocking the permeabilization with a FCS solution in Triton X 0.01%; and (v) adding DAPI for 15 min. The pictures were then taken with Cytation 1 and processed using Gen5™ Micro-plate Data Collection and Analysis Software v. 3.12 (BioTek^®^ Instruments Inc., Winooski, VT, USA). For a quantitative analysis of the Eug impact at the cellular level, the cell apoptosis index (AI) was calculated using the previously described formula [76]:

$$\mathrm{AI}=\frac{Number\;of\;apoptotic\;cells}{Total\;number\;of\;cells}\times 100$$

In accordance with the published protocols, the nuclear morphology was evaluated using ImageJ software v.6 [77].

### 4.6. Gene Expression Ratio

To investigate the potential cell-death mechanism induced by Eug 0.5 mM in SAOS-2 and Detroit-562 cells, the expression of pro-apoptotic (Bax, Bad) and anti-apoptotic (Bcl-2) markers was evaluated using the real-time reverse transcription–polymerase chain reaction (RT-PCR) technique after 72 h of treatment. RNA extraction was performed using the Quick-RNA Miniprep Kit (Zymo Research, Irvine, CA, USA), and the amount of extracted RNA was measured with the DS-11 spectrophotometer (DeNovix, Wilmington, DE, USA). The cDNA was produced using the Maxima^®^ First Strand cDNA Synthesis Kit, and the RT-qPCR quantification was performed using a Quant Studio 5 real-time PCR system (Thermo Fisher Scientific, Inc., Waltham, MA, USA) and Power SYBR-Green PCR Master Mix (Thermo Fisher Scientific, Inc., Waltham, MA, USA).

### 4.7. Caspase-3/7, -8, and -9 Activity

The activity of caspases 3/7, 8, and 9 was determined after the SAOS-2 and Detroit-562 cells’ 72 h treatment with Eug 0.5 mM, using the Caspase-Glo Kit provided by Promega, Madison, WI, USA. The plates were first equilibrated at room temperature for 30 min, then, the Caspase-Glo reagent (100 μL/well) was added. The plates were shaken for 30 s on a plate shaker and maintained at room temperature for another 2 h. Finally, the luminescence was measured on Cytation 5 (BioTek Instruments Inc., Winooski, VT, USA).

### 4.8. The RealTime-Glo™ Annexin V Apoptosis and Necrosis Assay

To confirm the apoptotic effect of Eug 0.5 mM in Saos-2 and Detroit-562 cells, the RealTime-Glo™ Annexin V Apoptosis and Necrosis Assay was performed according to the manufacturer’s protocol. In brief, the cells were cultured in solid white bottom 96-well plates (Costar^®^ 3917) and treated with Eug. The prepared 2X Detection Reagent that contained Annexin V NanoBiT™ Substrate, Annexin V-SmBiT, Annexin V-LgBiT, CaCl_2_, and Necrosis Detection Reagent was added in each well and the plates were shaken for 30 s at 500 rpm. The luminescence and fluorescence (excitation 485 ± 20 nm, and emission 525 ± 30 nm) were recorded at different time points (0, 6, 12, 24, 48, and 72 h post-treatment) using Cytation 5. Background signals (from no-cell wells) that were subtracted from the wells that contained cells.

### 4.9. Hen’s Egg Test—Chorioallantoic Membrane (HET-CAM)

The irritant and toxic potential of Eug (1 mM) at the vascular level was assessed by performing the HET-CAM assay, as previously described [78]. In brief, several steps were followed: (i) chicken eggs (*Gallus gallus domesticus*) were washed, disinfected with ethanol 70%, and incubated; (ii) on day 4 after incubation, a perforation was made in each egg to extract 6 to 7 mL of albumen; (iii) on the next day of incubation, a window was cut in the upper part of the eggs to facilitate the visualization of the blood vessels, and was covered using adhesive tape; (iv) on day 10 of incubation, 600 µL of each sample (negative control—H_2_O, positive control—SDS 1%, and Eug 1 mM) were added to the chorioallantoic membrane; (v) the compounds’ ability to produce hemorrhage (H), vascular lysis (L), and coagulation was monitored for 5 min, taking photos (using the Discovery 8 Stereomicroscope from Zeiss, Göttingen, Germany and the Zeiss Axio CAM 105 color camera) prior to the addition of the samples (T0) and at the end of the 5 min period (T5); and (vi) the irritation score (IS) was finally determined for each sample, according to the following formula:

$$\mathrm{IS}=5\times \frac{301-H}{300}+7\times \frac{301-L}{300}+9\times \frac{301-C}{300},$$

where IS = irritation score, H—the time at which the hemorrhage was observed, L—the time at which vascular lysis occurred and C—the time at which intravascular coagulation was established.

### 4.10. Statistical Analysis

The results of the study were expressed as means ± standard deviation (SD), the statistical differences between the groups being compared by means of the one-way ANOVA method. GraphPad Prism version 9.5.1 software was used for statistical analysis. (GraphPad Software, San Diego, CA, USA, www.graphpad.com). The statistically significant differences between the data were labeled with * (* *p* < 0.1; ** *p* < 0.01; *** *p* < 0.001; **** *p* < 0.0001).

## 5. Conclusions

By exploring the potential antitumor activity of Eug in vitro against osteosarcoma cells (SAOS-2) and oropharynx squamous carcinoma cells (Detroit-562), and by examining its potential vascular toxicity in ovo, the present study concludes the following novel findings: (i) Eug exerts a concentration-dependent cytotoxicity against osteosarcoma (SAOS-2) and oropharyngeal squamous cancer (Detroit-562) cells by causing a significant reduction in cell viability, as well as changes in cellular morphology and confluence, especially at high concentrations (≥ 0.5 mM); (ii) Eug (0.5 mM) exerts its cytotoxic effect by inducing apoptosis-like effects in the tested cell lines, which was indicated by the nuclear condensation and fragmentation and elevation in the expression of Bax and Bad markers, accompanied by the reduction in Bcl-2 expression; increase in the activity of caspase-3/7, -8, and -9; and PS externalization to the outer cell surface before the membrane loss of integrity caused by secondary necrosis; and (iii) Eug exerts a moderate irritation effect at the CAM level at the highest investigated concentration (1 mM). Based on these observations, additional studies are required to confirm the applicability of Eug in the treatment of these particular cancer types.

## Figures and Tables

**Table 1:** Irritation score values for the negative control (H_2_O), positive control (SDS 1%), and Eug 1 mM.

	H_2_O	SDS 1%	Eug 1 mM
IS	0.15	19.87	1.69
tH	300	21	300
tL	299	17	278
tC	298	15	263

IS = irritation score, tH, tL, and tC—the time at which hemorrhage (H), vascular lysis (L), and intravascular coagulation were established (C).

## Data Availability

The data presented in this study are available on request from the corresponding author.

## References

[1] Cragg G.M., Pezzuto J.M. (2016). Natural Products as a Vital Source for the Discovery of Cancer Chemotherapeutic and Chemopreventive Agents. Med. Princ. Pract..

[2] Tobeiha M., Rajabi A., Raisi A., Mohajeri M., Yazdi S.M., Davoodvandi A., Aslanbeigi F., Vaziri M.S., Hamblin M.R., Mirzaei H. (2021). Potential of Natural Products in Osteosarcoma Treatment: Focus on Molecular Mechanisms. Biomed. Pharmacother..

[3] Bhalla Y., Gupta V.K., Jaitak V. (2013). Anticancer Activity of Essential Oils: A Review. J. Sci. Food Agric..

[4] Blowman K., Magalhães M., Lemos M.F.L., Cabral C., Pires I.M. (2018). Anticancer Properties of Essential Oils and Other Natural Products. Evid.-Based Complement. Altern. Med..

[5] Barboza J.N., da Silva Maia Bezerra Filho C., Silva R.O., Medeiros J.V.R., de Sousa D.P. (2018). An Overview on the Anti-Inflammatory Potential and Antioxidant Profile of Eugenol. Oxid. Med. Cell Longev..

[6] Park S.H., Sim Y.B., Lee J.K., Kim S.M., Kang Y.J., Jung J.S., Suh H.W. (2011). The Analgesic Effects and Mechanisms of Orally Administered Eugenol. Arch. Pharm. Res..

[7] Benencia F., Courrèges M.C. (2000). In Vitro and in Vivo Activity of Eugenol on Human Herpesvirus. Phytother. Res..

[8] Gülçin I. (2011). Antioxidant Activity of Eugenol: A Structure–Activity Relationship Study. J. Med. Food..

[9] Zari A.T., Zari T.A., Hakeem K.R. (2021). Anticancer Properties of Eugenol: A Review. Molecules.

[10] Khalil A.A., Rahman U.U., Khan M.R., Sahar A., Mehmood T., Khan M. (2017). Essential Oil Eugenol: Sources, Extraction Techniques and Nutraceutical Perspectives. RSC Adv..

[11] Fadilah F., Yanuar A., Arsianti A., Andrajati R. (2017). Phenylpropanoids, Eugenol Scaffold, and Its Derivatives as Anticancer. Asian J. Pharm. Clin. Res..

[12] Begum S.N., Ray A.S., Rahaman C.H. (2022). A Comprehensive and Systematic Review on Potential Anticancer Activities of Eugenol: From Pre-Clinical Evidence to Molecular Mechanisms of Action. Phytomedicine.

[13] Beird H.C., Bielack S.S., Flanagan A.M., Gill J., Heymann D., Janeway K.A., Livingston J.A., Roberts R.D., Strauss S.J., Gorlick R. (2022). Osteosarcoma. Nat. Rev. Dis. Primers.

[14] Mirabello L., Troisi R.J., Savage S.A. (2009). International Osteosarcoma Incidence Patterns in Children and Adolescents, Middle Ages, and Elderly Persons. Int. J. Cancer.

[15] Zhang J., Walsh M.F., Wu G., Edmonson M.N., Gruber T.A., Easton J., Hedges D., Ma X., Zhou X., Yergeau D.A. (2015). Germline Mutations in Predisposition Genes in Pediatric Cancer. N. Engl. J. Med..

[16] Cundy T. (2018). Paget’s Disease of Bone. Metabolism.

[17] Tucker M.A., D’Angio G.J., Boice J.D., Strong L.C., Li F.P., Stovall M., Stone B.J., Green D.M., Lombardi F., Newton W. (1987). Bone Sarcomas Linked to Radiotherapy and Chemotherapy in Children. N. Engl. J. Med..

[18] Martins-Neves S.R., Sampaio-Ribeiro G., Gomes C.M.F. (2023). Self-Renewal and Pluripotency in Osteosarcoma Stem Cells’ Chemoresistance: Notch, Hedgehog, and Wnt/β-Catenin Interplay with Embryonic Markers. Int. J. Mol. Sci..

[19] Ferrari D., Moneghini L., Allevi F., Bulfamante G., Biglioli F. (2017). Osteosarcoma of the Jaw: Classification, Diagnosis and Treatment. Osteosarcoma-Biology, Behavior and Mechanisms.

[20] Zhao X., Wu Q., Gong X., Liu J., Ma Y. (2021). Osteosarcoma: A Review of Current and Future Therapeutic Approaches. Biomed. Eng. Online.

[21] Smeland S., Bielack S.S., Whelan J., Bernstein M., Hogendoorn P., Krailo M.D., Gorlick R., Janeway K.A., Ingleby F.C., Anninga J. (2019). Survival and Prognosis with Osteosarcoma: Outcomes in More than 2000 Patients in the EURAMOS-1 (European and American Osteosarcoma Study) Cohort. Eur. J. Cancer.

[22] Jamal Z., Anjum F. (2023). Oropharyngeal Squamous Cell Carcinoma. StatPearls.

[23] Panarese I., Aquino G., Ronchi A., Longo F., Montella M., Cozzolino I., Roccuzzo G., Colella G., Caraglia M., Franco R. (2019). Oral and Oropharyngeal Squamous Cell Carcinoma: Prognostic and Predictive Parameters in the Etiopathogenetic Route. Expert Rev. Anticancer Ther..

[24] Fauzi F.H., Hamzan N.I., Rahman N.A., Suraiya S., Mohamad S. (2020). Detection of Human Papillomavirus in Oropharyngeal Squamous Cell Carcinoma. J. Zhejiang Univ. Sci. B.

[25] Stern P.L., Dalianis T. (2021). Oropharyngeal Squamous Cell Carcinoma Treatment in the Era of Immune Checkpoint Inhibitors. Viruses.

[26] Lee T.Y., Tseng Y.H. (2020). The Potential of Phytochemicals in Oral Cancer Prevention and Therapy: A Review of the Evidence. Biomolecules.

[27] Padhy I., Paul P., Sharma T., Banerjee S., Mondal A. (2022). Molecular Mechanisms of Action of Eugenol in Cancer: Recent Trends and Advancement. Life.

[28] Bray F., Ferlay J., Soerjomataram I., Siegel R.L., Torre L.A., Jemal A. (2018). Global Cancer Statistics 2018: GLOBOCAN Estimates of Incidence and Mortality Worldwide for 36 Cancers in 185 Countries. CA Cancer J Clin..

[29] Costello L., Toner M., Pierse D., Stassen L.F.A. (2021). Osteosarcoma (Osteogenic Sarcoma) of the Jaws Presenting in General Dental Practice—A Series of Four Cases. Br. Dent. J..

[30] Golusiński W., Golusińska-Kardach E. (2019). Current Role of Surgery in the Management of Oropharyngeal Cancer. Front. Oncol..

[31] Ali Abdalla Y.O., Subramaniam B., Nyamathulla S., Shamsuddin N., Arshad N.M., Mun K.S., Awang K., Nagoor N.H. (2022). Natural Products for Cancer Therapy: A Review of Their Mechanism of Actions and Toxicity in the Past Decade. J. Trop. Med..

[32] Nisar M.F., Khadim M., Rafiq M., Chen J., Yang Y., Wan C.C. (2021). Pharmacological Properties and Health Benefits of Eugenol: A Comprehensive Review. Oxid. Med. Cell. Longev..

[33] Ulanowska M., Olas B. (2021). Biological Properties and Prospects for the Application of Eugenol—A Review. Int. J. Mol. Sci..

[34] Luiz De Sá Júnior P., Aparecida D., Câmara D., Santos Costa A., Luis J., Ruiz M., Levy D., Azevedo R.A., Fernanda K., Pasqualoto M. (2016). Apoptotic Effect of Eugenol Envolves G2/M Phase Abrogation Accompanied by Mitochondrial Damage and Clastogenic Effect on Cancer Cell in Vitro. Phytomedicine.

[35] Al Wafai R., El-Rabih W., Katerji M., Safi R., El Sabban M., El-Rifai O., Usta J. (2017). Chemosensitivity of MCF-7 Cells to Eugenol: Release of Cytochrome-c and Lactate Dehydrogenase. Sci. Rep..

[36] Ramazani E., YazdFazeli M., Ahmad Emami S., Mohtashami L., Javadi B., Asili J., Tayarani-Najaran Z. (2020). Protective Effects of Cinnamomum Verum, Cinnamomum Cassia and Cinnamaldehyde against 6-OHDA-Induced Apoptosis in PC12 Cells. Mol. Biol. Rep..

[37] Shin S.H., Park J.H., Kim G.C., Park B.S., Gil Y.G., Kim C.H. (2007). The Mechanism of Apoptosis Induced by Eugenol in Human Osteosarcoma Cells. J. Korean Assoc. Oral Maxillofac. Surg..

[38] Surducan D.A., Racea R.C., Cabuta M., Olariu I., Macasoi I., Rusu L.C., Chiriac S.D., Chioran D., Dinu S., Pricop M.O. (2023). Eugenol Induces Apoptosis in Tongue Squamous Carcinoma Cells by Mediating the Expression of Bcl-2 Family. Life.

[39] Racea R.C., Merghes P.E., Gurgus D., Macasoi I., Rusu L.C., Chioran D., Dinu S., Breban-Schwarzkopf D., Szuhanek C., Rivis M. (2023). Eugenol: In Vitro Characterization Of The Cytotoxic Profile At The Level Of Colorectal Carcinoma Cells. Farmacia.

[40] (2009). Biological Evaluation of Medical Devices—Part 5: Tests for In Vitro Cytotoxicity.

[41] Razak M.A.I.A., Hamid H.A., Othman R.N.I.R., Moktar S.A.M., Miskon A. (2020). Improved Drug Delivery System for Cancer Treatment by D-Glucose Conjugation with Eugenol From Natural Product. Curr. Drug Deliv..

[42] Rothzerg E., Pfaff A.L., Koks S. (2022). Innovative Approaches for Treatment of Osteosarcoma. Exp. Biol. Med..

[43] Wen Y., Grandis J.R. (2015). Emerging Drugs for Head and Neck Cancer. Expert Opin. Emerg. Drugs.

[44] Kis A.M., Macasoi I., Paul C., Radulescu M., Buzatu R., Watz C.G., Cheveresan A., Berceanu D., Pinzaru I., Dinu S. (2022). Methotrexate and Cetuximab—Biological Impact on Non-Tumorigenic Models: In Vitro and In Ovo Assessments. Medicina.

[45] Sramek M., Neradil J., Sterba J., Veselska R. (2016). Non-DHFR-Mediated Effects of Methotrexate in Osteosarcoma Cell Lines: Epigenetic Alterations and Enhanced Cell Differentiation. Cancer Cell Int..

[46] Valizadeh A., Shanehbandi D., Yousefi B., Soleimanpour J. (2022). Thymoquinone Potentiates Methotrexate Mediated-Apoptosis in Saos-2 Osteosarcoma Cell Line. Drug Res..

[47] Young N.R., Soneru C., Liu J., Grushko T.A., Hardeman A., Olopade O.I., Baum A., Solca F., Cohen E.E.W. (2015). Afatinib Efficacy against Squamous Cell Carcinoma of the Head and Neck Cell Lines in Vitro and in Vivo. Target Oncol..

[48] Lin S.R., Chang C.H., Hsu C.F., Tsai M.J., Cheng H., Leong M.K., Sung P.J., Chen J.C., Weng C.-F. (2020). Natural Compounds as Potential Adjuvants to Cancer Therapy: Preclinical Evidence. Br. J. Pharmacol..

[49] Duan Y., Huang X., Qiao B., Ma R., Li J. (2022). Eugenol Inhibits the Biological Activities of an Oral Squamous Cell Carcinoma Cell Line SCC9 via Targeting MIF. Anticancer Agents Med. Chem..

[50] Kim Y.H., Park B.S. (2015). The Effect of Eugenol on the Induction of Apoptosis in HSC-2 Human Oral Squamous Cell Carcinoma. Orig. Artic. J. Korean Soc. Dent. Hyg..

[51] Yoo C.-B., Han K.-T., Cho K.-S., Ha J., Park H.-J., Nam J.-H., Kil U.-H., Lee K.-T. (2005). Eugenol Isolated from the Essential Oil of Eugenia Caryophyllata Induces a Reactive Oxygen Species-Mediated Apoptosis in HL-60 Human Promyelocytic Leukemia Cells. Cancer Lett..

[52] das Chagas Pereira de Andrade F., Mendes A.N. (2020). Computational Analysis of Eugenol Inhibitory Activity in Lipoxygenase and Cyclooxygenase Pathways. Sci. Rep..

[53] Fangjun L., Zhijia Y. (2018). Tumor Suppressive Roles of Eugenol in Human Lung Cancer Cells. Thorac. Cancer.

[54] Fathy M., Fawzy M.A., Hintzsche H., Nikaido T., Dandekar T., Othman E.M. (2019). Eugenol Exerts Apoptotic Effect and Modulates the Sensitivity of HeLa Cells to Cisplatin and Radiation. Molecules.

[55] Ziegler U., Groscurth P. (2004). Morphological Features of Cell Death. Physiology.

[56] Menyhárt O., Harami-Papp H., Sukumar S., Schäfer R., Magnani L., de Barrios O., Győrffy B. (2016). Guidelines for the Selection of Functional Assays to Evaluate the Hallmarks of Cancer. Biochim. Biophys. Acta Rev. Cancer..

[57] Doonan F., Cotter T.G. (2008). Morphological Assessment of Apoptosis. Methods.

[58] Vidhya N., Devaraj N. (2011). Induction of Apoptosis by Eugenol in Human Breast Cancer Cell. Indian J. Exp. Biol..

[59] Das A., Harshadha K., Dhinesh Kannan S.K., Hari Raj K., Jayaprakash B. (2018). Evaluation of Therapeutic Potential of Eugenol-A Natural Derivative of *Syzygium aromaticum* on Cervical Cancer. Asian Pac. J. Cancer Prev..

[60] Mandelkow R., GüMBEL D., Ahrend H., Kaul A., Zimmermann U., Burchardt M., Stope M.B. (2017). Detection and Quantification of Nuclear Morphology Changes in Apoptotic Cells by Fluorescence Microscopy and Subsequent Analysis of Visualized Fluorescent Signals. Anticancer Res..

[61] Permatasari H.K., Effendi A.B., Qhabibi F.R., Fawwaz F., Dominique A. (2021). Eugenol Isolated from *Syzygium aromaticum* Inhibits HeLa Cancer Cell Migration by Altering Epithelial-Mesenchymal Transition Protein Regulators. J. Appl. Pharm. Sci..

[62] Kim G.C., Choi D.S., Lim J.S., Jeong H.C., Kim I.R., Lee M.H., Park B.S. (2016). Caspases-Dependent Apoptosis in Human Melanoma Cell by Eugenol. Korean J. Anat..

[63] Lindenboim L., Zohar H., Worman H.J., Stein R. (2020). The Nuclear Envelope: Target and Mediator of the Apoptotic Process. Cell Death Discov..

[64] Islam S.S., Al-Sharif I., Sultan A., Al-Mazrou A., Remmal A., Aboussekhra A. (2018). Eugenol Potentiates Cisplatin Anti-Cancer Activity through Inhibition of ALDH-Positive Breast Cancer Stem Cells and the NF-ΚB Signaling Pathway. Mol. Carcinog..

[65] Hemaiswarya S., Doble M. (2013). Combination of Phenylpropanoids with 5-Fluorouracil as Anti-Cancer Agents against Human Cervical Cancer (HeLa) Cell Line. Phytomedicine.

[66] Banerjee S., Panda C.K., Das S. (2006). Clove (*Syzygium aromaticum* L.), a Potential Chemopreventive Agent for Lung Cancer. Carcinogenesis.

[67] Sarkar A., Bhattacharjee S., Mandal D.P. (2015). Induction of Apoptosis by Eugenol and Capsaicin in Human Gastric Cancer AGS Cells-Elucidating the Role of P53. Asian Pac. J. Cancer Prev..

[68] Dhani S., Zhao Y., Zhivotovsky B. (2021). A Long Way to Go: Caspase Inhibitors in Clinical Use. Cell Death Dis..

[69] Kupcho K., Shultz J., Hurst R., Hartnett J., Zhou W., Machleidt T., Grailer J., Worzella T., Riss T., Lazar D. (2019). A Real-Time, Bioluminescent Annexin V Assay for the Assessment of Apoptosis. Apoptosis.

[70] RealTime-GloTM Annexin V Apoptosis Assay|Annexin V Staining|Apoptosis Assay. https://www.promega.ro/products/cell-health-assays/apoptosis-assays/realtime-glo-annexin-v-apoptosis-assay/?catNum=JA1011.

[71] Winter G., Koch A.B.F., Löffler J., Jelezko F., Lindén M., Li H., Abaei A., Zuo Z., Beer A.J., Rasche V. (2020). In Vivo PET/MRI Imaging of the Chorioallantoic Membrane. Front. Phys..

[72] Budai P., Kormos É., Buda I., Somody G., Lehel J. (2021). Comparative Evaluation of HET-CAM and ICE Methods for Objective Assessment of Ocular Irritation Caused by Selected Pesticide Products. Toxicol. Vitr..

[73] Ahmad N., Jalees Ahmad F., Bedi S., Sharma S., Umar S., Azam Ansari M. (2019). A Novel Nanoformulation Development of Eugenol and Their Treatment in Inflammation and Periodontitis. Saudi Pharm. J..

[74] Thanekar D., Dhodi J., Gawali N., Raju A., Deshpande P., Degani M., Juvekar A. (2016). Evaluation of Antitumor and Anti-Angiogenic Activity of Bioactive Compounds from Cinnamomum Tamala: In Vitro, in Vivo and in Silico Approach. S. Afr. J. Bot..

[75] Gad El-Hak H., Gerges M. (2018). Evaluating the teratogenic effect of Eugenol in the development of the chick embryos. J. Biol. Stud..

[76] Gag O., Macasoi I., Pinzaru I., Dinu S., Popovici R., Cosoroaba M.R., Buzatu R., Cabuta M., Chiriac S.D. (2023). In Vitro Assessment of the Impact of Ultraviolet B Radiation on Oral Healthy and Tumor Cells. Photonics.

[77] Eidet J.R., Pasovic L., Maria R., Jackson C.J., Utheim T.P. (2014). Objective Assessment of Changes in Nuclear Morphology and Cell Distribution Following Induction of Apoptosis. Diagn. Pathol..

[78] Rednic R., Macasoi I., Pinzaru I., Dehelean C.A., Tomescu M.C., Susan M., Feier H. (2022). Pharmaco-Toxicological Assessment of the Combined Cytotoxic Effects of Digoxin and Betulinic Acid in Melanoma Cells. Life.

